# Proper Management for Massive Penetrating Chest Trauma Narrowly Missing Major Organs: A Case Report

**DOI:** 10.1155/cris/1663332

**Published:** 2026-08-06

**Authors:** Jacek Szulc, Konstantinos Kostopanagiotou, Joanna Markopoulou, Tomasz Grodzki, Małgorzata Edyta Wojtyś

**Affiliations:** ^1^ Student Scientific Club of Department of Thoracic Surgery and Transplantation, Pomeranian Medical University in Szczecin, Szczecin 70-204, Poland, pum.edu.pl; ^2^ Thoracic Surgery Department, National and Kapodistrian University of Athens School of Medicine, Attikon University General Hospital, Chaidari 12462, Greece, attikonhospital.gr; ^3^ Department of Thoracic Surgery and Transplantation, Pomeranian Medical University in Szczecin, Alfreda Sokołowskiego 11, Szczecin 70-891, Poland, pum.edu.pl

**Keywords:** case report, chest trauma, impalement, penetrating injury, thoracotomy

## Abstract

Chest impalement is a rare type of injury resulting from penetration by a large foreign object that remains in the body. Each case of chest impalement is unique, and the outcome largely depends on the trajectory of the foreign object. Here we present the case of a 48‐year‐old male who accidentally fell from the rooftop of his house (from a height of several meters), onto a scaffolding tube, which penetrated through the 10th left posterior intercostal space into his chest. Upon transport of the patient to the trauma center, computed tomography (CT) surprisingly revealed no significant damage to the internal organs. The foreign body was successfully removed by left posterolateral thoracotomy, and the postoperative course was uneventful. A good outcome following a chest injury requires timely transport to the trauma center, and multidisciplinary management by an experienced team. In the presented case, the foreign object’s trajectory did not cause damage to the internal organs, which was also an important factor contributing to the good outcome.

## 1. Introduction

Chest trauma accounts for ~25% of injury‐related deaths and is the third most common cause of death in patients with polytrauma [1]. In the latest data from the German Trauma Register DGU (from 2022–2024), a thoracic injury is present in 46.1% of patients with severe injuries (abbreviated injury scale > 3+) [2]. Within civilian populations, traumatic chest injuries are most commonly caused by traffic accidents, followed by ground‐level falls, falls from heights, assault, and industrial accidents [3]. Based on the mechanism of injury, chest trauma can be characterized as blunt or penetrating, with blunt trauma being more frequent (70%–80%). Compared to blunt trauma, penetrating chest trauma more commonly requires surgical intervention and is associated with higher all‐cause mortality [4, 5]. In severe polytrauma cases, time is critical, and fast assessment and intervention are essential for patient survival [5]. In general, injury severity is dictated by the amount of energy transferred to the human body; however, in a penetrating injury, the type of penetrating object or projectile is also an important factor. In a stab wound, the damage is limited to the trajectory of the blade, whereas in a gunshot wound, the tissues adjacent to the wound cavity may also be significantly damaged by the kinetic energy of the bullet. Moreover, particularly in impalement injuries, the outcome depends on the track of the foreign object [1].

The aim of the present report is to describe a rare case of massive penetrating chest trauma and to discuss proper management of such cases. The unusual trajectory of the foreign object was probably a main factor affecting the patient’s favorable outcome despite the initial presentation.

## 2. Case Presentation

**Figure 1:**
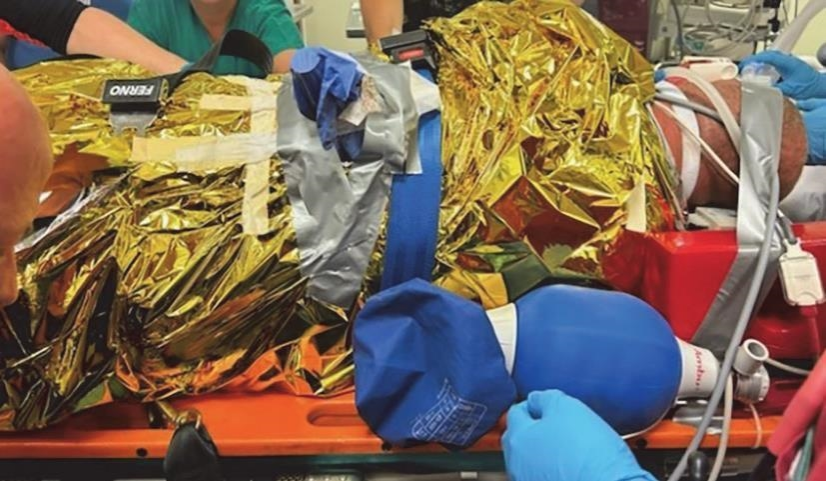
Impalement injury treatment requires proper stabilization of the object, to avoid dislocation and further intracavitary injury, while maintaining proper airway access and thermoprotection.

A 48‐year‐old male, without underlying health conditions, was working on the rooftop of his house (at a height of approximately 4 m) and accidentally fell from the rooftop onto a scaffolding tube, which penetrated through his back. The firefighters who responded to the scene had to cut the scaffolding tube, because it was embedded in the ground. There was no visible blood loss, and the patient was hemodynamically stable. However, the foreign body had an open lumen and was causing open pneumothorax, prompting chest tube insertion. Given the massive character of the trauma, the helicopter emergency service team intubated the patient at the scene. The patient was then transported to the hospital under controlled conditions (Figure 1).

**Figure 2:**
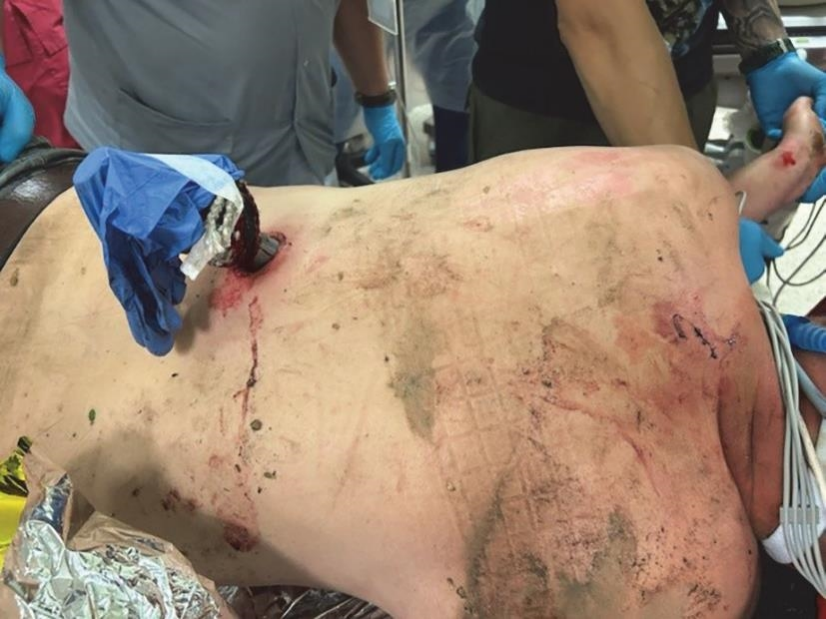
During physical examination, the body must be fully exposed to identify all potential injuries, which may include fractures, soft‐tissue hematomas, and lacerations. Note the external occlusion of the tube lumen, to prevent open pneumothorax.

To avoid secondary damage, the penetrating scaffolding tube was stabilized (Figure 2). Upon presentation at our hospital, the penetrating object exhibited transmitted pulsation, suggesting that it was abutting the heart. The patient’s blood was drawn for lab tests and cross‐matching, in case a transfusion was needed.

**Figure 3:**
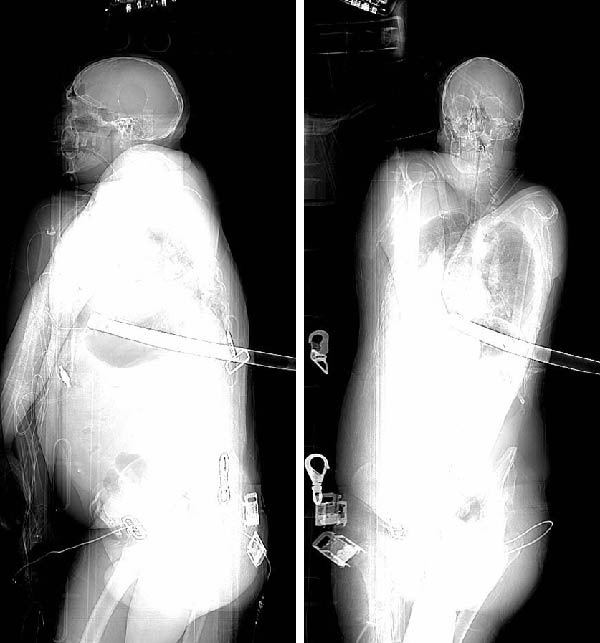
A computed tomography (CT) localizer radiograph was obtained before a full CT scan, and shows the potential scale of the injuries. An impaled object should be removed after complete CT scan with contrast, and under controlled conditions. Note that emergency thoracotomy without CT is indicated in selected unstable patients.

**Figure 4:**
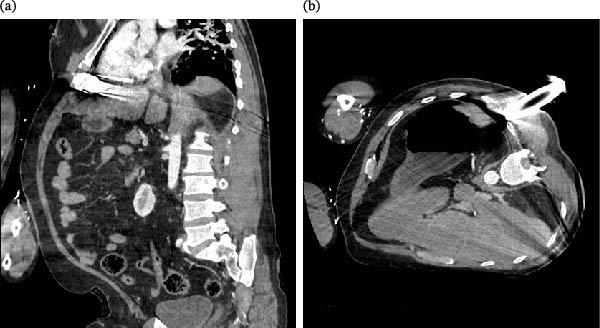
(a and b) Trauma computed tomography (CT) scan excluded cardiac, pericardial, mediastinal, tracheal, esophageal, or parenchymal damage amenable to treatment. The diaphragm is best assessed intraoperatively. The image shows that the metallic tube entered the chest through the 10th posterior left intercostal space, and is lying on the left hemidiaphragm adjacent to the heart and lung.

**Figure 5:**
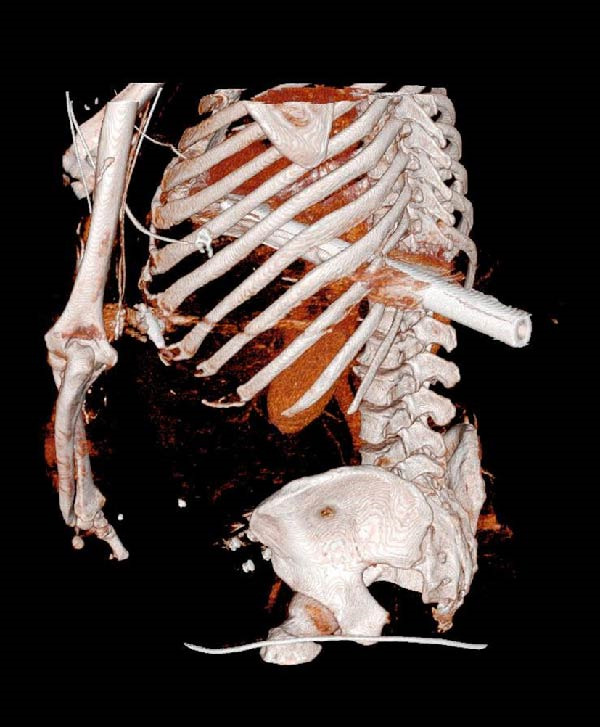
Three‐dimensional computed tomography reconstruction.

After the initial physical examination, polytrauma computed tomography (CT) was performed to assess any injuries to the internal organs and to guide selection of an appropriate operative approach. CT revealed a metallic tube, with a 30‐mm diameter, entering the chest through the 10th left posterior intercostal space. The tube was lying on the left hemidiaphragm, adjacent to the heart and lungs, and there was no evidence of injury to these organs. The distal end of the tube reached the anterior chest wall. Posterior fractures of the left 9th and 10th ribs were observed. There was no evidence of injuries to the great vessels or abdominal organs (Figures 3, 4a,b, and 5).

**Figure 6:**
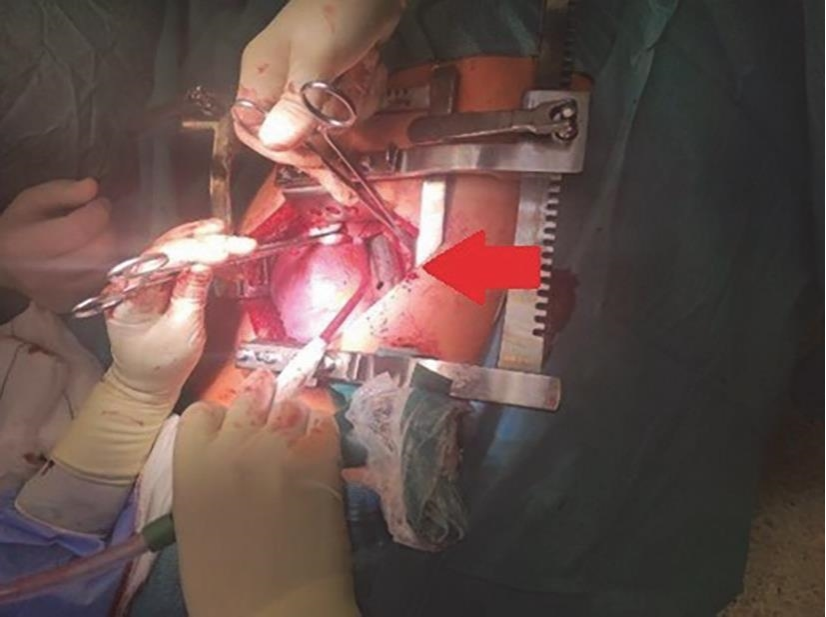
Standard posterolateral thoracotomy, at the appropriate level, enables full inspection of the pleural cavity and a range of maneuvers. The red arrow is pointed at the foreign object.

**Figure 7:**
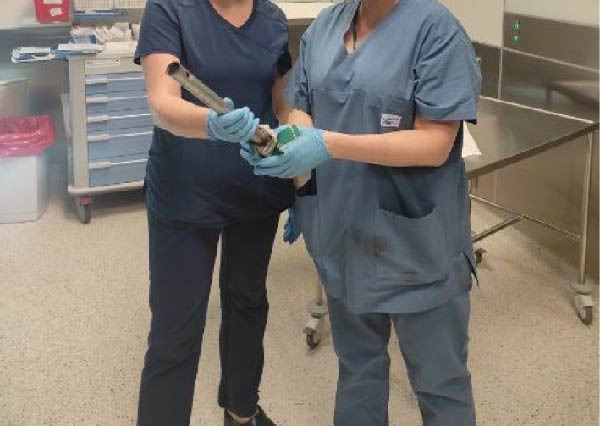
The size of the penetrating object could have potentially damaged a variety of internal structures. Moreover, its hollow design could have been catastrophic in the event of exsanguination or massive pneumothorax.

The patient was transferred to the operating room for removal of the foreign object. Posterolateral left thoracotomy was performed, with the incision line below the 9th rib, above the foreign body intrusion (Figure 6). During the operation, the foreign object was removed, and *z*‐sutures were used to repair a diaphragm rupture measuring ~4 cm in length (Figure 7). No other injuries were observed. The thoracic cavity was rinsed with povidone‐iodine solution, and then the chest was closed in layers, with two chest tubes maintained in the left pleural cavity.

**Figure 8:**
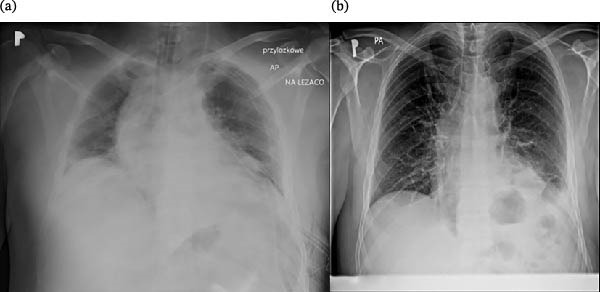
(a) Chest x‐ray performed on the first postoperative day. (b) Follow‐up chest x‐ray at the 2‐month follow‐up.

After surgery, the patient was transferred to the intensive care unit. He was mechanically ventilated, received tetanus toxoid, and was started on noradrenaline infusion, as well as piperacillin/tazobactam combined with metronidazole (this intravenous regimen was continued for the next 7 days). Chest x‐ray was performed on the first postoperative day (Figure 8a). On the second postoperative day, the patient was extubated and noradrenaline was stopped. On the fourth postoperative day, the patient was transferred to the thoracic surgery department. The first chest drain was removed on the eighth postoperative day and the second chest drain on the ninth postoperative day. Blood transfusions were not required during hospitalization. Ten days after surgery, the patient was discharged from the hospital and was satisfied with the treatment. At the 2‐month follow‐up, the patient was doing well, reporting only mild typical post‐thoracotomy chest wall pain, which had resolved by the next check‐up. The chest x‐ray revealed expanded lungs (Figure 8b), and the patient denied any signs of deteriorated respiratory function, such as dyspnea. No neuropathic pain was present. The patient eventually returned to his preaccident functional status. At 33 months after the operation, we contacted the patient to obtain consent for publication; he reported that he was doing well and with no pain.

## 3. Discussion

Our patient’s injury can be classified as type I impalement. Of the two categories of impalement injuries, type I is more common and occurs when a moving body falls into an immobile object, as in the presented case, while type II impalement involves the reverse: a moving object penetrating an immobile body [6]. In the present case, the foreign object followed a unique trajectory, penetrating the body in‐between major structures, i.e., the heart and lung, without causing any evident injury to these organs. This trajectory was likely the most important factor influencing the positive outcome, though not the only one, as exemplified by reports of patients who survive massive chest trauma despite a foreign body traversing the heart, lungs, and large vessels [7, 8]. One should not underestimate the importance of proper and immediate management by an experienced trauma team.

Chest trauma management can be divided into three stages: prehospital life support, emergency room life support, and surgical intervention [5]. Initial patient assessment at the scene should include the identification of immediately life‐threatening injuries, including airway obstruction, flail chest, open pneumothorax, tension pneumothorax, massive hemothorax, cardiac injuries, and rupture of the great vessels [9]. This initial on‐scene assessment is predominantly based on physical examination and vital signs monitoring but can also include the use of small mobile ultrasound by trained personnel. Patients with reduced or absent breath sounds on auscultation and signs of cardiorespiratory distress should be suspected of tension pneumothorax and managed by chest tube placement. A chest tube can also be placed for pleural hematoma treatment. While 28–32 French (1F = 1⁄3 mm) chest tubes are most commonly used, 24F chest tubes may be used in patients with narrow intercostal spaces, and 36F chest tubes may be useful for draining a dense hematoma. Smaller bore tubes are associated with less pain but may result in incomplete drainage and subsequent empyema. In the presently reported case, a chest tube was placed by paramedics at the scene of the accident [1, 10–12]. Securing the airways is another important aspect of chest trauma management, which can be achieved through rapid sequence intubation in instances of major chest trauma. Patients with chest trauma also commonly require oxygen therapy and adequate fluid therapy. Crystalloids are recommended during the prehospital phase, although their use should be limited to avoid acidosis and dilutional coagulopathy. Appropriate pain management must also be provided. The latest edition of the Advanced Trauma Life Support Guide recommends multimodal pain relief—using drugs with different mechanisms of action, both orally and intravenously—which allows avoidance of side effects and the use of lower doses [1, 13–15]. The general principles of managing patients with thoracic trauma also apply to cases of chest impalement; however, chest impalement is a rare and unique type of injury that often requires an individualized approach and immediate decision‐making. Importantly, in cases of impalement, the foreign object should not be removed until surgery to prevent secondary injuries and fatal hemorrhaging [16].

In patients with chest trauma, the first‐choice imaging modalities are chest x‐ray and extended focused assessment with sonography for trauma. This also applies in cases of impalement; however, imaging should not excessively delay surgery when needed. In patients who have been initially stabilized, CT can also be utilized. In our present case, the use of CT allowed us to exclude major injury to the heart, lung, and great vessels and to identify the trajectory of the foreign object, which was crucial for selecting a surgical approach [12, 17, 18].

Impaled foreign bodies should be removed under direct vision, which enables a detailed assessment of the injuries and prevents secondary damage [6]. Thoracotomy is the main operative approach in such situations. In our current case, left posterolateral thoracotomy was selected based on the CT scan findings and the location of the entry wound. Sternotomy may sometimes be used when required due to the injury location [19]. In our case, the thoracic surgeon observed a diaphragmatic rupture during surgery that had not been detected on CT. Penetrating diaphragm injuries are often intraoperatively diagnosed as multidetector CT has only a 74% sensitivity for identifying these injuries [20]. There are several specific signs of diaphragmatic rupture on a CT scan, including collar sign, dependent viscera sign, and herniation of abdominal viscera in the thoracic cavity; however, none of these were present in our current case [21]. Notably, the rupture in our case was only ~4 cm in length, likely contributing to the difficulty of preoperative diagnosis. This highlights the importance of thoroughly inspecting the thoracic cavity in patients with thoracic trauma. It is important to detect and repair any diaphragm rupture because such injuries tend to enlarge due to constant diaphragm motion, potentially leading to herniation [22, 23]. Our patient also exhibited fractures of the 9th and 10^th^ ribs, which were conservatively treated with pain management and pulmonary rehabilitation—the latter being essential to avoid pneumonia. Conservative therapy is the mainstay of treatment for rib fractures. Standard pain management can sometimes be supplemented with intercostal nerve blockade, paravertebral blockade, or even epidural anesthesia, and surgical stabilization may be necessary in selected cases [1].

Thoracic trauma can lead to numerous complications, with the most severe including acute respiratory distress syndrome and multiple organ failure. Furthermore, infectious complications are common in chest trauma patients, with pneumonia developing in 5%–40% of cases [1]. In particular, penetrating chest injuries are associated with a high risk of contamination, which increases the risk of infectious complications. Hence, the postoperative care of patients after penetrating chest trauma should include antibiotics to prevent infectious complications [24]. Numerous studies have demonstrated the efficacy of antibiotic prophylaxis for reducing the infection risk in patients with penetrating chest injury necessitating chest tube placement (OR 0.28, 0.14–0.57) [25]. In the present case, antibiotic prophylaxis was administered using piperacillin/tazobactam combined with metronidazole. Broad‐spectrum dual coverage was selected due to soil contamination of the penetrating foreign body and the massive character of the injury. Tetanus prophylaxis must also be remembered in such cases [26].

To summarize, impalement injuries are rare and pose a challenge for thoracic surgeons due to the uniqueness of each case. The good outcome in the present case was the result of a combination of factors—including the trajectory of the foreign object, stabilization of the foreign body, timely transport to the trauma center, and multidisciplinary management by an experienced team.

**Nomenclature**
CT: Computed tomography.

## Author Contributions

**Małgorzata Edyta Wojtyś:** conceptualization, methodology, formal analysis, investigation, resources, data curation, writing – original draft preparation, writing – review and editing, visualization. **Jacek Szulc**: conceptualization, methodology, formal analysis, investigation, data curation, writing – original draft preparation, supervision. **Konstantinos Kostopanagiotou**: formal analysis, investigation, data curation, writing – original draft preparation, writing – review and editing, visualization, supervision. **Tomasz Grodzki**: formal analysis, investigation, resources, data curation, writing – original draft preparation, writing – review and editing, visualization, supervision. **Joanna Markopoulou**: formal analysis, investigation, data curation, writing – original draft preparation.

## Funding

This research received no external funding.

## Disclosure

All authors have read and agreed to the published version of the manuscript.

## Ethics Statement

The study does not require ethical approval, according to the Bioethical Committee of Pomeranian Medical University (KB.008.092.2026), from 12 March 2026.

## Consent

Written informed consent has been obtained from the patient to publish this paper.

## Conflicts of Interest

The authors declare no conflicts of interest.

## Data Availability

The data presented in this study are available within the article.
